# How oral cancer is taught: a scoping review

**DOI:** 10.3389/froh.2026.1841566

**Published:** 2026-07-30

**Authors:** Lalima Tiwari, Omar Kujan

**Affiliations:** Oral and Maxillofacial Biology & Diseases Research Stream, UWA Dental School, The University of Western Australia, Nedlands, WA, Australia

**Keywords:** dental education, education, oral cancer, oral cancer education, oral potentially malignant disorders

## Abstract

**Objectives:**

This scoping review aims to examine the current modes of delivery of oral cancer education amongst health care practitioners and students. It also seeks to identify challenges in teaching and gaps within existing educational approaches.

**Methods:**

Studies examining the mode of delivery of oral cancer education among any health care practitioners, university students, or training programs were included in the literature search across bibliographic databases.

**Results:**

Thirty-nine studies were found to be eligible for inclusion. A variety of modes of oral cancer education delivery were identified, including oral presentations, hands-on training in head and neck examination, case-based discussions and problem-based learning approaches, clinical observation, biopsy performance, and a range of structured educational models. All teaching methods demonstrated improvements in oral cancer knowledge compared with baseline, to varying degrees. The time allocated to oral cancer education ranged from 0 to 50 h, with a median duration of 2 h.

**Conclusions:**

Consensus on the most effective mode of delivery is lacking; however, findings across all studies indicate that any form of oral cancer education intervention is preferable to none. Furthermore, there is an urgent need to increase the amount of time devoted to oral cancer education across health curricula.

## Introduction

Early detection of oral squamous cell carcinoma (OSCC) and oral potentially malignant disorders (OPMD) is well established as a critical factor in reducing morbidity and mortality, and in improving long-term survival and quality of life (1). Despite this, challenges in the early diagnosis of OPMDs and OSCC continue to arise due to patient, professional, referral and scheduling delays (2, 3). While there has been an improvement in patients seeking advice from primary health care professionals, the interval time for health professionals making a diagnosis has not significantly improved (1, 3). Current data indicates that approximately 53% of patients with OSCC initially present to their medical general practitioner (GP), 42% to their general dental practitioner (GDP), and 5% directly to a dental or medical specialist. These patterns highlight the critical need for both medical and dental professionals to be competent in identifying high-risk oral mucosal cases and facilitating prompt referral to an appropriate specialist (2, 3). Globally, a significant proportion of diagnostic delays has been attributed to professional knowledge of oral cancer, with many studies around the world underscoring the importance of strengthening practitioner knowledge to improve early detection and diagnosis of oral cancer and overall patient care (4–7).

In Australia, Idrees et al. reported on the lack of adequate knowledge in identifying OPMDs amongst dental health professionals and dental students, noting an overall accuracy rate of 65.9% in the identification of oral mucosal lesions (4). In a Brazilian study, 64.6% and 51.4% of participating dentists and dental students, respectively, did not feel able to identify oral cancer (8). Furthermore, 52.7% of dental students and 67.1% of dentists from the same study did not believe that they received appropriate or any training during their dental education, including palpation of lymph nodes and identification of lymphadenopathies associated with oral cancer (8). Brailo et al. evaluated six university-based oral medicine units in Europe and identified an urgent need to increase clinical exposure of dental students to OPMDs to increase student confidence in OPMD detection and management (9). These findings were similarly replicated amongst medical students in the United Kingdom, whereby 95% of graduating medical students believed they were poorly informed on oral cancer (10). Further, a systematic review involving 3409 GPs demonstrated limited knowledge on emerging oral cancer risk factors and reported a range of 38%–94% of GPs feeling unsure about diagnosing oral cancer (5). Collectively, these findings underline the need to examine how clinicians are currently trained on oral cancer.

Traditionally, dental and medical curricula integrate didactic (online and face-to-face teaching), laboratory, and clinical skill development to foster clinical competence in the graduating student. However, few studies have evaluated the effectiveness of the mode of delivery specifically in the context of oral cancer education. There is also a growing body of evidence demonstrating the benefits of immersive virtual reality (IVR) and artificial intelligence in health education, such as increased engagement and personalising learning suggestions based on learners' learning status, preferences, and individual attributes (11, 12). However, these technologies have not been fully evaluated within the context of oral cancer education.

Given the current inadequacies in oral cancer and OPMD education for both dental and medical practitioners, this scoping review aims to examine the current modes of delivery of oral cancer education amongst health care practitioners and students. The review also seeks to identify challenges in teaching and gaps within existing educational approaches.

## Materials and methods

### Protocol and registration

Before conducting the scoping review, the research questions were formulated using the PCC Framework and the scoping review was carried out in accordance with the Preferred Reporting Items for Systematic Reviews and Meta-Analyses extension for Scoping Reviews (PRISMA-ScR) (13). The review protocol was registered on the Open Science Framework osf.io/p7rvm, Registration DOI 10.17605/OSF.IO/B3TQP.

For the scoping review, health care practitioners and health care students that underwent education on oral cancer or OPMDs were the populations of interest. The concept is “current modes of delivery and effectiveness of oral cancer education” and context is “university curriculum or continuing education programs”.

The research question includes

- What are the current modes of delivery of oral cancer education amongst health care practitioners and students?
- How effective are the current modes of delivery in oral cancer education?
- What are the gaps within educational approaches including technology used in oral cancer education?

### Data sources and search strategy

Electronic databases Medline, Web of Science, Embase and Scopus were searched until September 30th, 2025, using a combination of keywords and “MESH terms” in consultation with a librarian outlined in Table 1. In addition, grey literature was searched including Google Scholar and references were hand-checked from bibliographies in relevant articles and included in this review (Supplementary Table 1).

**Table 1:** Search strategy.

No.	Search statement using mesh terms and keywords
1	mouth diseases/or lichen planus, oral/or lip diseases/or mouth neoplasms/or oral submucous fibrosis/or oral ulcer/or tongue diseases/
2	curriculum/or education, nonprofessional/or education, predental/or education, premedical/or education, professional/or education, continuing/or education, dental/or education, medical/or education, medical, continuing/or education, medical, graduate/or education, medical, undergraduate/or teaching rounds/ or education, nursing/or education, pharmacy/or education, public health professional/
3	(oral cancer or mouth cancer or oral lichen planus or lip cancer or mouth neoplasm* or oral submucous fibrosis or oral ulcer or tongue cancer or tongue neoplasm*).tw.
4	1 or 3
5	[(education or training) adj5 (professional* or dentist* or general practitioner or doctor)].tw.
6	2 or 5
7	4 and 6

### Selection process

One screener (L.T) independently screened the citation titles and abstracts against the inclusion criteria, using EndNote21 to identify the papers eligible for full text analysis. A second screener (O.K) verified the results and resolved disagreements about study eligibility. Full-text articles were independently screened by both authors.

The inclusion criteria used in the selection of literature for this review were as follows:

- Published studies focusing on the mode of delivery of oral cancer education (including oral potentially malignant disorders) amongst any health care practitioners, university students or university programs.
- All study designs, including randomised controlled trials, cohort studies, case-control studies, cross-sectional studies, reviews and case reports.
- Publications in English.
- Studies published within the last 30 years.

Exclusion Criteria:

- Studies that are not focused on oral cancer or OPMDs.
- Studies not related to education or curriculum.
- Non-English publications as translation resources were not available.

### Types of participants

Any health care practitioner or university student (medical, dental, allied health) that underwent education on oral cancer or OPMDs.

### Types of interventions

Studies for inclusion had to evaluate the mode of delivery of oral cancer or OPMD education either in a university setting or a continuing education program.

### Types of outcome measures

#### Primary outcomes

Primary outcome measures for this review focused on identifying and categorising the current modes of delivery of oral cancer education and evaluating the efficacy of modes of delivery of oral cancer education.

#### Secondary outcomes

Secondary outcomes were to identify challenges in teaching, gaps in the current research, and suggest areas for future studies.

### Data extraction

A standardised data extraction form was developed. Key information was extracted, including study characteristics, mode of education delivery, participants involved, learning outcomes, measures used to assess learning outcomes, effectiveness of oral cancer education, limitations of the study and future recommendations.

## Results

### Study selection

**Figure 1:**
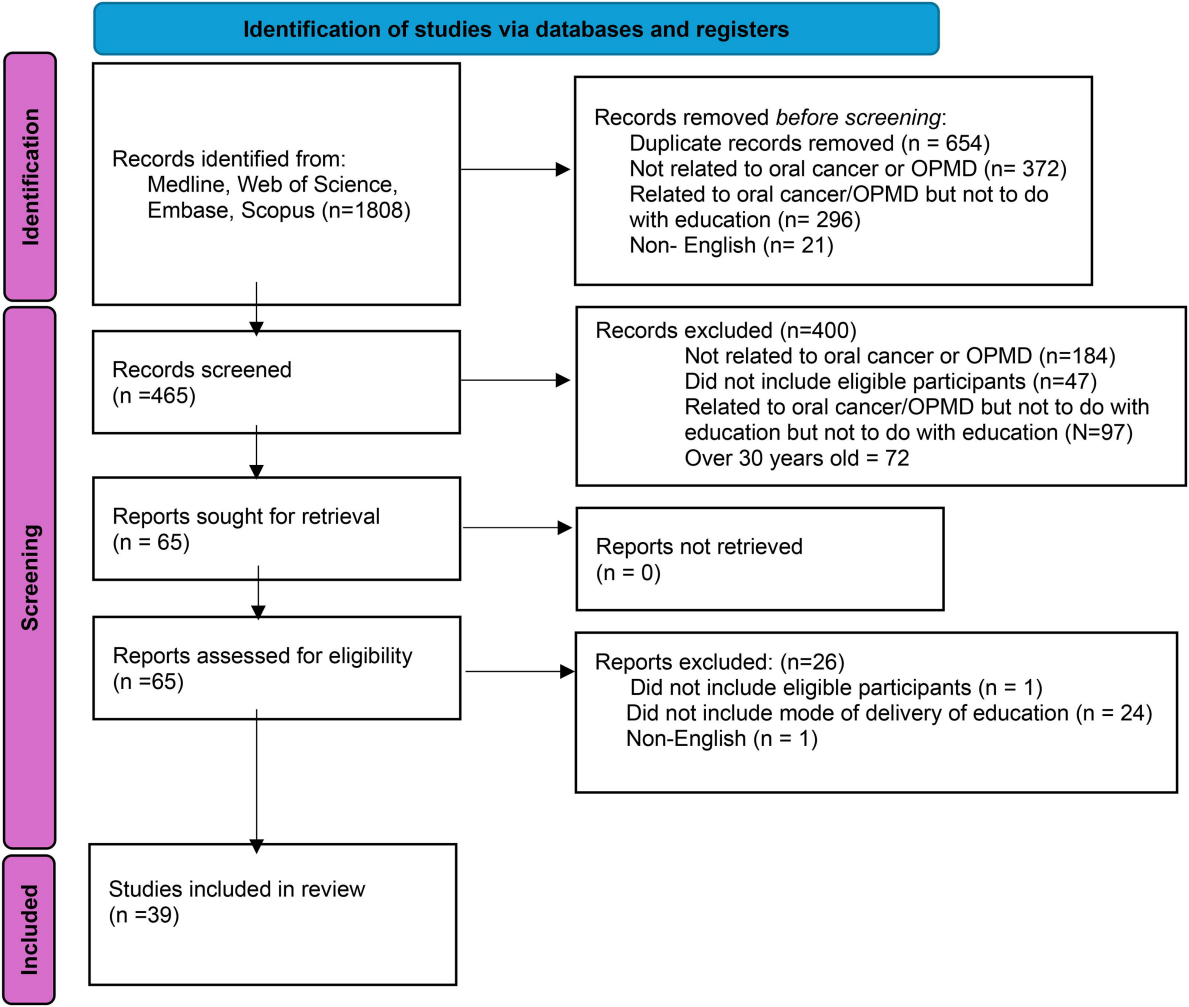
PRISMA flow diagram of screened studies.

A total of 1,808 studies were screened by title and abstract, with 65 full-text articles assessed for eligibility, and only 39 studies met the inclusion criteria (Figure 1). For each study, data was extracted using standardised forms and summarised based on the primary and secondary outcomes (Table 2). Out of the 39 studies, 20 studies were cross-sectional in design (14–33), 16 studies were interventional (pre-test, post-test) studies (34–49), 1 study was retrospective (50), and 2 studies were review articles (51, 52). 25 (64.1%) studies focused on oral cancer education within the dental profession only (dentists, dental students, dental hygienists or dental programs) (17, 19, 22, 23, 25, 27, 28, 30, 32–39, 41, 43–50), while 8 (20.5%) studies involved only the medical profession (medical practitioners, medical students, or medical programs) (14–16, 18, 20, 24, 26, 52). Two (5.1%) studies included only nursing students or nursing programs (21, 40), and 4 (10.2%) studies assessed oral cancer education amongst multiple health professionals including a combination of pharmacists, nurses, medical doctors, midwives, public health professionals and nutritionists (29, 31, 42, 51).

**Table 2:** Summary of mode of delivery of oral cancer education assessed and key findings of the included studies.

No	Author, Publication Year	Sample size, Participation rate (%)	Population type assessed	Mode of delivery of oral cancer education assessed	Outcomes assessed	Key Findings from study
1	Abbott et al. (2018)	8 (44%)	Dean or Head of Australian Medical Schools	Number of hours spent teaching oral cancer Hands on oral health training	Associated with dental school Total teaching hours of oral cancer Hands-on oral health training	Mean hours dedicated to oral cancer teaching was <1 h, median 0.22 h 6 medical schools reported <1 h of oral cancer teaching. 3 medical schools reported no oral cancer teaching No medical schools provided hands-on oral health training
2	Ahluwalia et al. (2016)	132 (24.7%)	GP Training Programme Directors in UK	Open format to assess current mode of delivery provided to GP trainees in 2012–2013 in UK	Oral health teaching and training delivered between August 2012 and July 2013	27.3% reported that none of their GP trainees undertook hospital posts to oral health 71.2% reported that their programme did not provide any oral health education for trainees through structured educational activities 18.8% had formal structured oral health education Teaching sessions were mainly led by ENT or OMFS Format of teaching primarily lectures or seminars Two respondents described trainee directed learning sessions Preferred teaching methods: tutors with expertise in oral health and oral cancer, e-learning programmes, problem-based learning
3	Ahluwalia et al. (1998)	86 (63.2%)	Medical schools in UK	Open assessment of how health history standard and physical examination pertaining to oral cancer detection taught in UK medical schools	Health history variables: sun exposure, dysphagia, difficulty speaking or hoarseness, sore throat, oral sores that do not heal, alcohol use, tobacco use Physical examination variables: evaluation of anatomical areas of oral cavity, lymph node examination, use of a light source	Combined score: Most schools scored less than optimal score in health and physical examination. Mean score of 4.39 out of 11 was noted for health history. Mean score was 7.32 out of 11 for physical examination. 95% of schools discussed tobacco use. 12.9% asked about sun exposure. 56.3% discussed oral cancer signs and symptoms. 28.6% of schools examined all anatomical areas of oral cavity. 52% of schools assessed high risk site including floor of mouth. Only 31% advised to protrude tongue during examination. 50% included lymph node palpation.
4	Awojobi et al. (2016)	39 completed pre-test (63%), 33 completed post-test (37%), 23 completed 2 week follow up (30%)	General dentists in UK	Training session that lasted 1.5 h, divided into four sections: a brief update on oral cancer, an introduction to the oral cancer communication guide, learning activities including watching video of the guide being used in practice, role play and feedback	Oral cancer screening behaviours Current practice regarding talking about cancer Barriers to communication Self-efficacy Intentions to discuss oral cancer prevention with high-risk patients	No statistically significant difference in oral cancer screening pre-training vs. post-training and at follow-up. Significant increase in dentists reporting that they informed their patients being screened for oral cancer after training and at follow-up. No significant increase in using the term “cancer” from pre-training to follow-up. Significant increase in discussing topics of oral cancer including cancer sites, signs and symptoms, early detection, risk factors, reducing risk factors, role or regular attendance, with patients from pre-training to follow-up. Training resulted in reduction in number of perceived barriers in communication e.g., “talking about oral cancer may frighten my patients”. Training resulted in increase in self-efficacy and was maintained at follow-up.
5	Braun et al. (2021)	221 (97.4%)	General dentists in Brazil	Continuing Education Activity including 4 h of oral presentation on: oral cancer epidemiology, risk factors, clinical characteristics and reasons for delay in diagnosis, impact on mortality rate, promote awareness. Evaluated set of simulated cases supported with clinical photographs for assessment of diagnostic abilities Participate in discussion regarding diagnosis for each case-based learning approach	Impact on daily clinical practice Frequency of oral examination and lesions detected Experience of oral cancer cases Number of continuing education programs attended on oral cancer Self-efficacy on diagnosis and treatment of oral lesions Self-efficacy on performing biopsy.	Statistically significant association between self-efficacy to diagnose/biopsy/treat oral lesions and the perception of time spent on theoretical lectures and practical learning as adequate. Dentists who perform oral mucosal examination more frequently perceived less difficulty in diagnosing and treating oral lesions. 88.9% dentists who indicated that they detect and diagnose oral lesions frequently attended oral cancer continuing education than dentists who did not attend continuing education. Difficulty to diagnose oral lesions was associated with less experience with oral cancer diagnosis (*p* < 0.1)
6	Cannick et al. (2007)	106, 8 underwent OSCE	1st and 2nd year dental students	Intervention group observed dental faculty members demonstrate proper head and neck examination techniques and how to counsel a standardized patient about tobacco use cessation based on PRECEDE-PROCEED model in addition to existing curriculum Control group received usual instruction in oral cancer prevention and detection as determined by dental curriculum including traditional lecture and PBL approach	Pretest and 6-month post-test assessment on oral cancer and tobacco cessation knowledge, opinions and behaviours, competency in head and neck examination and tobacco cessation counselling	Second year dental students in intervention group demonstrated higher knowledge on oral cancer compared to control group. No group difference noted for 1st year students. PRECEDE-PROCEED theoretical model can be applied as a comprehensive methodology for designing, implementing, and evaluating an oral cancer prevention and early detection intervention. Traditional lecture and PBL approach, reinforced by observing faculty members demonstrate head and neck examination and tobacco cessation can improve detection skills.
7	Cardoso et al. (2022)	442	General dentists	Modular Object—Oriented Dynamic Learning Environment (Moodle) Course: comprised of 50 h, distributed into 10 unites for 3 months. Problem based learning was used as teaching methodology. Learning objects released weekly, structure based on flipped classroom model. Participants had access to reading material, watch video in which tutors presented diagnostic reasoning, artificial environment (virtual learning object) to practice the diagnosis of oral lesions available. Single choice quiz at end of each unit to reinforce topic.	Pretest/Post test done to evaluate impact of course on participants' diagnostic abilities.	Moodle showed positive impact for all evaluated parameters. 13.4% increase post-test for correct classification of lesions, 10% increase in correct diagnoses. Confidence to manage benign lesions increased by 40%.
8	Carlisle et al. (2021)	N/A	Pharmacy and Dentistry Students	Interprofessional education strategies between undergraduate pharmacy and dentistry students	Interprofessional education strategies	Most frequently reported method was case-based activities/case studies to engage in oral cancer education Clinical setting: Pharmacy and dentistry students together in dental clinic allowed opportunity for increased identification of high-risk patients and tobacco smokers and increase uptake of tobacco cessation by patients.
9	Carter et al. (2010)	30 (66%)	Medical school curriculum coordinators in UK	Examination of how oral cancer education was taught in UK medical schools	Oral health education provided Teaching on oral anatomy and pathology Prevention, investigation and treatment of oral disease Identification of important risk factors	OMFS and ENT surgeons most involved specialties involved in oral health/oral cancer education Time spent teaching oral cancer varied from 1 h to 4–5-week course 5 medical schools offered oral cancer related study modules Teaching of oral cancer risk factors was satisfactory (70% teaching smoking) 12 schools provided examination of patients 13 schools provided clinical demonstration 11 schools provided lectures Other modes of teaching included: clinical skills, counselling, shadowing, problem-based learning, small group teaching, special study modules 40% of medical schools included oral cancer history taking in student assessment 35% included clinical examination of mouth 55% included clinical examination of neck
10	Clark et al. (2013)	30 (37%)	Dental Students	Video only instruction on theoretical oral cancer education and clinical examination technique Faculty-led hands-on instruction on theoretical oral cancer education and clinical examination technique Video and hands on instruction on theoretical oral cancer education and clinical examination technique	Effectiveness of interventions on skills training associated with oral and pharyngeal examination Confidence and sense of adequacy before and after intervention	All students improved in written examination scores All students significantly improved in general and clinical technique knowledge Students who received both video and hands-on training improved the most Students who received both video and hands on training significantly better from students who received video-only training
11	Fasbinder et al. (2014)	98 (92%)	1st and 2nd year dental students	Grand Rounds presentation by dental students with assigned faculty member	Explore how interested students were in learning about topics, Subjective student evaluation of the grand rounds course components and outcomes	Most students positively evaluated grand rounds presentation on oral cancer and requested it as ongoing topic for grand rounds. Students appreciated interdisciplinary nature of presentations. Students disliked course-related issues such as assignments, homework, length and timing of the class, assessment that followed the presentations
12	Gaballah et al. (2022)	49 (73.1%)	Dental interns and Junior dentists	Continuing education activity in form of interactive half day workshop hosted by experienced oral and maxillofacial surgeon. Workshop comprised lectures, case discussion using problem-based learning approach to identify oral mucosal changes, diagnosing various oral lesions, recognizing risk factors and features that suggestive of oral malignancy.	Changes in accuracy of diagnosis of oral mucosal lesions Changes in suspicion of malignancy of oral mucosal lesions	The education intervention significantly improved the overall diagnostic accuracy. Improvement most evident in ability of dentists to diagnose pathological changes either in form of benign lesions or OPMDs. No significance improvement observed in diagnosing cases with either normal variation or malignancies. CE activity improved overall suspicion of malignancy
13	Glossop et al. (2024)	14	Systematic review discussing undergraduate medical students at any point of their course in UK	Oral cancer and dental education teaching Teaching interventions	Oral cancer and dental education teaching Teaching intervention	Knowledge of OMFS and oral cancer can be increased effectively in a short space of time with an online series. 61% of medical students had the opportunity to examine oral lesions in patients compared with 88% dental students
14	Gomes et al. (2024)	113	Dentists and undergraduate dental students in Brazil	Continuing education activity including 90 min oral presentation and discussion with oral medicine professors on SPIKE protocol for communicating bad news.	Experience and perceptions of dentists and dental students on delivering cancer diagnoses	Only 22% of participants had inclusion of curriculum content related to this domain Proportion of participants who felt confident and highly confident after completing the CEA increased from 33.6% and 7.1%, respectively to 54% and 15.9%. Similar outcome observed regarding confidence in addressing the family of the patient who is about to receive oral cancer diagnoses.
15	Hamid et al. (2018)	200	Medical students in United Kingdom	Open assessment of medical curriculum	Ability to refer to head and neck specialities Exposure to OMFS Knowledge of oral cancer	Exposure to oral cancer was significantly lower than exposure to throat cancer across all teaching domains including clinics, lectures, tutorials. More students who had rotation weeks and lectures on OMFS were more likely to refer patients with lesions of the mouth and tongue to the specialty
16	Haresaku et al. (2024)	105	First year nursing students	Lecture program included 45 min lecture on oral assessment. Practical program included 30 min oral assessment with simulators.	Confidence in oral assessment Perceptions regarding oral assessment	Nursing students' perception statistically higher in assessment of oral cancer after the practical programme than after the lecture programme. Assessing the need for dental referral was highest after the practical programme, and lowest at baseline Confidence levels were higher in all assessment categories after both the lecture and practical programmes
17	Heler et al. (2025)	47 (46%)	Nursing educational institutions	Open assessment of dental and oral health curriculum within nursing educational institutions	Nursing educators' knowledge confidence on dental concerns Nursing educators' perspective on nursing graduates' knowledge about oral health complications Nursing educators' perspective on challenges to incorporate dental and oral health education into the nursing curriculum	48.9% noted dental screening was not covered in curriculum 25.5% noted implementing education regarding oral cancer and oral lesions screening 80.9% of participants noted that future nurses should be educated about oral cancer and disease prevention
18	Ilguy et al. (2014)	55 (100%)	4th year undergraduate dental students	Lecture based learning group taught in conference manner in classroom, using lectures addressing various oral disease topics Case based learning groups: groups of 8 students, devoted discussion of clinical cases in oral diseases. No formal lectures were planned for this portion.	Clinical decision making based on SOLO taxonomy	Students who were taught with case-based learning methodology had the highest scores at the final two levels of the SOLO taxonomy compared to students taught with the lecture-based learning.
19	Lee et al. (2022)	180 (92%)	Dental hygienists	Questionnaire assessing teaching methods of oral cancer education, contents of the curriculum, adequacy of education	Awareness of the importance of oral cancer risk factors Knowledge of oral cancer risk factors Confidence in oral cancer education	75% of participants were aware of the importance of assessing risk factors of oral cancer. 51% performed assessments for oral cancer risk 52.8% thought lack of knowledge was main factor that prevented them from conducting effective assessments for oral cancer risk among themselves Knowledge of erythroplakia as more malignant than leukoplakia had the lowest percentage of correct answers. 48% received oral cancer education, 91% received during university training 81% reported supplemental oral cancer education and training was necessary 74% willing to participate in additional educational activities Educational content received: general symptoms of oral cancer, site of oral cancer, risk factors of oral cancer. 29.4% and 36.5% received education on oral cancer screening method and how to prevent oral cancer respectively.
20	LeHew et al. (2009)	8	Medical professionals	1-day training program in two sessions including presentation on oral cancer epidemiology and early detection Clinical training in palpation of head and neck, Instruction on risk factors.	Evaluate clinicians' training and estimation of how it would affect future practice	All participants intend to change their clinical practice as a result of the training. All participants found training on early detection of oral cancer to be worthwhile and would examine oral cavities and palpate the necks of tobacco users more often.
21	Macedo et al. (2025)	199	Dental students	Educational training received including: Performing biopsies Clinical examination Education type received	Demographic characteristics of participants Specific knowledge of clinical-epidemiologic characteristics and personal opinions on general oral cancer issues	66.3% of students did not receive training to perform biopsy procedure 84.4% of students thought their lymph node palpation training was inadequate 47.7% reported that they never attended an additional course on oral cancer, although 97.5% were interested in such courses 44.7% of students believed that dentists' knowledge regarding oral cancer was insufficient
22	McCann et al. (2005)	48 physicians 21 medical, dental and postgraduate deans (72%, 100%, 83%)	A&E physicians in United Kingdom and Deans of 29 medical schools in UK	Current teaching on oral anatomy and pathology Stage at which training was provided Professional background of the teachers	Current teaching on oral anatomy and pathology Prevention of oral disease Stage at which training was provided Professional background of the teachers	71% taught normal oral anatomy, in 1st and 2nd year 52% taught oral pathology, Clinical 3rd and 4th year 29% taught prevention of oral disease in preclinical years 56% of UK dental schools were involved in teaching medical undergraduates 38% of dental schools were consulted on the content of local medical course
23	Miller et al. (2011)	50	Dental Students	Smoking and alcohol module consisted of fifteen to twenty PowerPoint slides along with a continuous oral narration (by a professional narrator) describing and expanding upon the text and bullet points. The average time to complete each module was approximately fifteen minutes, although the modules were designed to allow the viewer to control the pace of the presentation.	Knowledge of tobacco use, oral health, and oral cancer risk and attitude towards screening for alcohol use	Both training modules resulted in meaningful improvement in dental students' knowledge Significant increase in overall proportion of correct responses comparing pre and post test results for tobacco and alcohol module (92% and 88%) Greatest improvement was for assessing knowledge of oral cancer risks in smokers (35% pre vs. 96% post) Module resulted in statistically significant increases in intention to screen patients for alcohol use and in how comfortable students would feel in asking patients about alcohol use
24	Morelatto et al. (2022)	60	Dentists, Dental Students	“Stick your tongue out” Theoretical and practical courses were organised to train dentists in detection of oral cancer lesions. Clinical records analysed for time of diagnosis. Stages III and IV defined as advanced, Stage I and II as early	Delay in diagnosis of oral cancer: patient delay, professional delay, system delay	Pre-intervention, 27% of oral cancer cases were diagnosed at initial stages, post-treatment period, early diagnosis increased to 40% Improvement especially noted in diagnosis of tumours located on the gums, floor of mouth and oropharynx. Lip cancer diagnosed early in both periods. Pre-treatment, biopsy only advised in 6%, post-treatment increased to 23.3% Pre-treatment period showed that professional delay more evident at early stages
25	Nethan et al. (2019)	40 (83%)	Health care professionals including medical doctors, dentists, nurses, midwives, general nurse, public health professionals	ECHO training module: brief didactic presentation via Zoom, extending for about 20–30 min, on a specific topic, presented by one of the team experts at the hub or an external expert in the concerned area. The didactics comprised PowerPoint presentations on a variety of topics covering the different facets of oral cancer screening, including sessions on tobacco cessation. This was followed by a brief discussion or a round of Q&A among the presenters and the participants. HCPs presented a case (new/ongoing) each on an OPMD or oral cancer and shared their personal experience	Knowledge gain regarding oral cancer screening and tobacco cessation	Significant knowledge gain among participants regarding oral cancer screening and tobacco cessation, rise in score to 7.4 compared to 6.7 in pre-evaluation Training showed significant impact on knowledge regarding best tobacco cessation drug
26	Nethan et al. (2023)	52	Dentists	14-week curriculum covering aspects of oral cancer screening and tobacco cessation. Each online session started with an expert-led didactic followed by a question-and-answer session every week, Thereafter, participants presented cases. Participants successfully completing the online course (*n* = 8/52) attended a one-day workshop that involved a revision of all the topics covered during the online training followed by hands-on training in oral visual examination and tobacco cessation.	• Assess the knowledge, attitude and practice of the participants regarding oral cancer screening and tobacco cessation after online training and after clinical exposure. • One year follow up survey to assess utilisation of skills.	Significant increase in overall mean knowledge score after programme and at weekly sessions Increase in confidence in oral cancer screening after online training
27	Ramanathan et al. (2024)	328	Final year dental students	OralDETECT training programme consists of series of tests, lectures, and corrective feedback sessions. Five structured lectures focusing on the target and non-target lesions. After the lectures, participants undertake the first post-test, followed by a discussion of the answers. This pattern repeats for the second post-test. Upon completion of the third post-test, participants are informed of their results.	Knowledge gained on conditions such as smoker's palate, angular cheilitis, betel chewer's mucosa, pseudomembranous candidiasis, and OPMD and oral cancer	Overall, the scores of the participants improved from the pre-test up until the post-test Trend of steady increase in mean scores followed by a slight decrease at the last assessment with face-to-face versus online approaches. Final score of the participants was significantly associated with the method of administration of the training programme
28	Ramirez-Amador et al. (2023)	22 deans (78.6%) 30 professors in workshop	Deans and directors of dentistry schools in Mexico and professors involved in teaching oral medicine	Workshop analysing and discussing main challenges of teaching OPMDs and oral cancer	Explore academic and professional profile of professors Identify strengths and opportunities proposed by leaders of OPM curricula. Obtain strategies to improve diagnosis by training future stomatologists.	77.3% included diagnosis of oral lesions as part of their curricula. 63.6% focused OPMD and oral cancer content in third year, from 6 months to 12 months duration. 86% used clinical cases, 73% case presentations, 86.4% written exams. 45% developed community activities for early detection of oral cancer, but 23% confirmed presence of oral medicine expert. 83% stated that OPM curriculum at undergraduate level required improvement. Teaching and practice of oral examination should be reinforced from 1st year of undergraduate levels. Oral examination should be prompted throughout the study courses. Problem based learning and clinical case presentations are most used teaching-learning strategies and must be encouraged to improve students' awareness. OPM professors must be trained to reorient the teaching-learning process from successful pedagogical models. Multidisciplinary activity is recommended.
29	Rankin et al. (1996)	198 (79%)	4th year undergraduate dental students	Assess whether students received experience in oral cancer topics, performed treatments or procedures during dental school, felt comfortable of skill in these areas	Intra oral biopsy Risk factors for oral cancer Management of oral complications of radiation and chemotherapy	54% of students observed biopsy of oral tissue 26% performed biopsy Students who had observed a biopsy or read a pathology report were significantly more likely to report that they were comfortable in performing a biopsy than those who had not. Significantly larger percentage of students who reported having performed a biopsy during dental school indicated that following graduation they intended to pursue advanced degree residency programs rather than general dentistry 81% were comfortable with their skills in counselling patients about cancer risk factors 83% were comfortable in recognising a malignant lesion Students who reported receiving instruction in prescribing nicotine replacement therapy were significantly more likely to do so in practice. 92% were taught neck examination
30	Roeschmann et al. (2022)	33	Dental students	Questionnaire based on Zimmermann et al. shows illustrations to define the lesion and includes patient medical history, tobacco use. Helps characterise location, size, type of lesion, surface and margin conditions	Agreement of stated suspected diagnosis with the actual pathological diagnosis Secondary outcome was difference between groups in terms of student descriptions	Use of structured questionnaire by Zimmerman et al., performed statistically significantly better for lesion description, lesion localisation, size, type of lesion, surface and margin
31	Roxo-Goncalves et al. (2017)	27 (54%)	Primary health care professionals including dentists Non- dentists include nurses, nutritionists	Web base self-instruction course on diagnosis of oral mucosal lesions, including case discussion activity. Lectures prepared on Microsoft Powerpoint software with accompanying audio recording.	Correct classification of oral mucosal lesions	Non-dentists and dentists showed a comparable sensitivity of 68.8 ± 11.1 and 63.7 ± 15.8, respectively. Specialists performed somewhat better; however, the difference was not statistically significant. Dentists and specialists showed higher specificity than nondentists. Most challenging cases for nondentists were OPMDs, and early SCC.
32	Seoane et al. (2011)	37 (100%)	5th year undergraduate dental students	Seminar workshop supported by simulated clinical cases (images and clinical records). Guidelines for referral of oral cancer published by Dental Council utilised.	Assess screening ability of control and experimental group for oral cancer and assess decision to refer.	The specificity in the diagnosis of oral cancer was significantly higher amongst students that underwent educational intervention. The proportion of oral cancers that were indicated for urgent referral by students in the experimental group was significantly higher. The number of oral cancers that received no indication for referral was superior in control group The use of referral guideline also lead to a significant increase in urgent referral of precancerous lesions/conditions.
33	Seoane et al. (1999)	44 (100%)	5th year undergraduate dental students	Pictorial manual with diagnostic criteria for oral red lesions. 2× one hour diagnostic seminar on oral erythroplakia and other red lesions.	Assess diagnostic accuracy of oral red lesions	Sensitivity, specificity and diagnostic concordance were all higher for experimental group, and differences were highly significant (*p* = 0.0001) for sensitivity and diagnostic concordance of oral red lesions.
34	Silva et al. (2016)	50	1st semester and 7th semester undergraduate dental students	Traditional teaching model: case study presentation to be solved in activities called “seminars”.	Compare percentages of correct answers, demonstrate concordance in percentages of correct answers.	Percentage of correct answers of seventh semester undergraduate students were significantly higher than first semester students. 91.6% vs. 64% of students of first semester correctly indicated smoking as the main factors in the development of the disease.
35	Tabrizi et al. (2020)	13	Medicine, Nursing, Dental Students	Program built on pedagogical model where students work together to solve health issues. Educational activities designed applying 4Ms concept to treating Alzheimer's disease, Oral Cancer, Parkinson's disease, Stroke. Faculty speakers discussed cancerous lesions. Interprofessional program where all activities presented in clinical setting.	Assess understanding of importance of oral health Assess knowledge about chronic condition including oral cancer Assess satisfaction regarding interdisciplinary teaching program	Students had a positive belief that oral health is an important aspect of overall health Students expressed that they did not get enough education on oral health Knowledge on oral cancer increased post intervention 7/11 students recommended bringing interprofessional course to their school
36	Thacker et al. (2015)	8 (66%)	Deans of associate-degree dental hygiene programmes	Evaluating oral cancer training concepts	Programme curriculum Teaching and learning methods	62.5% reported having specific curriculum on oral cancer 100% taught examination of oral cavity, oropharynx and base of tongue 100% taught palpation of nodes, buccal mucosa, floor of mouth and tongue. 37% taught palpation of tonsils and tonsillar pillars 100% taught tobacco as risk factor, 25% taught Betel nut as risk factor 0% taught alcohol counselling, compared to 100% tobacco cessation counselling 66% of students examine patients for oral cancer at every visit
37	Tomar et al	213	Dental students	School 1: took didactic course in oral oncology, received 17 h of lectures on various topics of oral cancer including epidemiology, aetiologic factors, histopathology, clinical appearance, clinical management and treatment of oral complications. School 2: Received only 50-minute introductory lecture, followed by self-paced course of ten weeks' duration	Knowledge comparison between self-study vs. didactic	Significantly higher scores noted in students with lecture plus reading material compared to self-directed teaching only.
38	Uti et al. (2006)	65 (89%)	Dental students	Evaluating exposure to oral cancer and OPMD Exposure to biopsy Knowledge on risk factors Management of malignant lesions	Knowledge and experience of dental students on management and prevention of oral cancer and OPMD	87.7% witnessed at least one patient with oral malignancy, 61.5% witnessed patient with premalignancy 89.2% and 69.2% thought tobacco smoking and alcohol consumption were the most important aetiological factors All respondents believed oral malignancy is always or sometimes associated with pain. 71.4% would routinely and immediately refer patients with malignant oral lesions to specialist 67.2% would do biopsy of malignant lesion routinely
39	Walsh et al. (2013)	543 (37%)	Dental hygienists	Lecture format involving 5-hour sessions. Two presenters conducted each course with 1 covering early detection, and other on tobacco cessation.	Knowledge, attitudes and practices related to oral cancer screening and tobacco cessation counselling.	Significant improvement in knowledge of contraindication to nicotine patch use for smoking cessation, asking tobacco users about age of tobacco initiation, Improvement in discussing roadblocks to quitting and identifying benefits of quitting. Significant improvement in performing visual exams of soft tissues, retracting the tongue to view lateral borders and in palpating the neck during oral cancer screening Significant improvement in informing patients of oral cancer screening procedure. No improvement in knowledge of risk factors from baseline to follow up

### Time spent on oral cancer education

15 studies reported on the duration of time spent on oral cancer education amongst health professionals, ranging from 0 h to 50 h, with the median duration of 2 h (14, 17–19, 24, 34, 36, 39–43, 46, 48, 49). Two studies reported that time spent on oral cancer education in medical schools varied significantly. Abbott et al., reported a median of 0.21 h and Carter at al., reported that time spent on oral cancer education ranged from 1 h to an 4–5 week integrated course (14, 18). One study reported on nursing students who received a 45 min lecture on oral assessment followed by 30 min oral assessment practical program with simulators (40). Studies within the dental profession spent a mean duration of 7.4 h (range 15 min to 50 h) (17, 19, 24, 34, 36, 39, 41–43, 46, 48, 49).

### Professionals involved in teaching

A variety of health professionals were involved in teaching oral cancer education including academics from medical, dental, dental hygiene, and nursing faculties. 8 studies specified the professional background of those involved in providing oral cancer education (15, 18–21, 26, 27, 52). Out of these studies, the main speciality involved in teaching oral cancer was Ear, Nose and Throat specialists and Oral and Maxillofacial Surgeons (62.5%) (15, 18–20, 52) and were primarily involved in teaching medical professionals (medical students, GP trainees) (15, 18, 20, 52). Two studies specified involvement of professors in oral medicine and oral pathology in teaching OPMDs and oral cancer (27, 39).

### Learning outcomes

**Figure 2:**
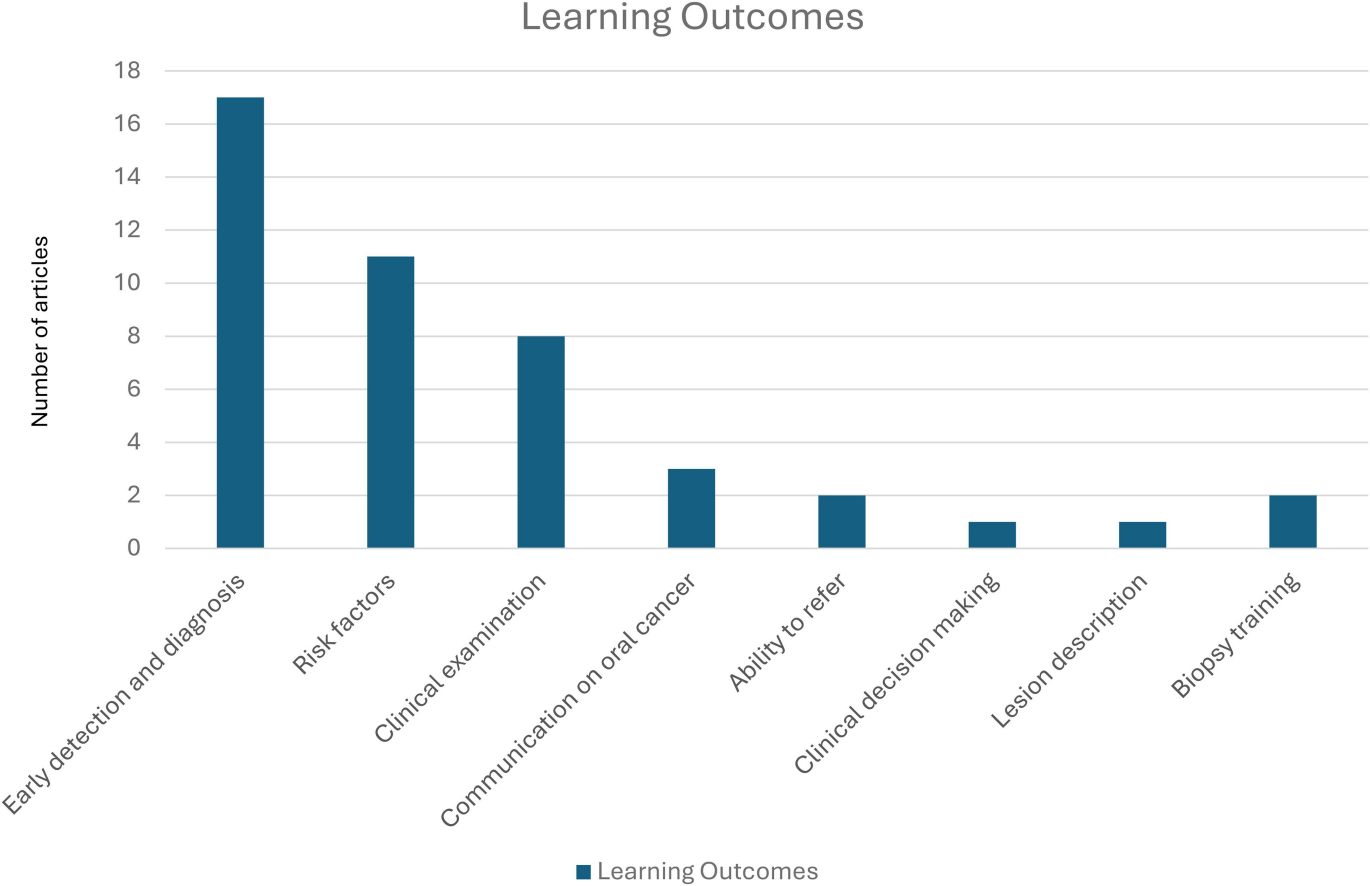
Learning outcomes discussed in the studies.

The most commonly taught learning outcome was early detection and diagnosis of oral cancer, with seventeen studies including topics on correct diagnosis and classification of oral lesions (Figure 2) (17–19, 21, 23, 24, 26, 29, 31, 33, 36, 38, 44, 46, 48, 50, 51). Knowledge on risk factors including sun exposure, alcohol use, tobacco use was discussed in 11 studies (15, 17, 18, 23, 28, 30, 32, 34, 41, 43, 49). Only 8 studies included oral cancer screening including clinical examination and palpation of lymph nodes (15, 25, 32, 34, 35, 37, 40, 42).

### Mode of delivery of oral cancer education

**Figure 3:**
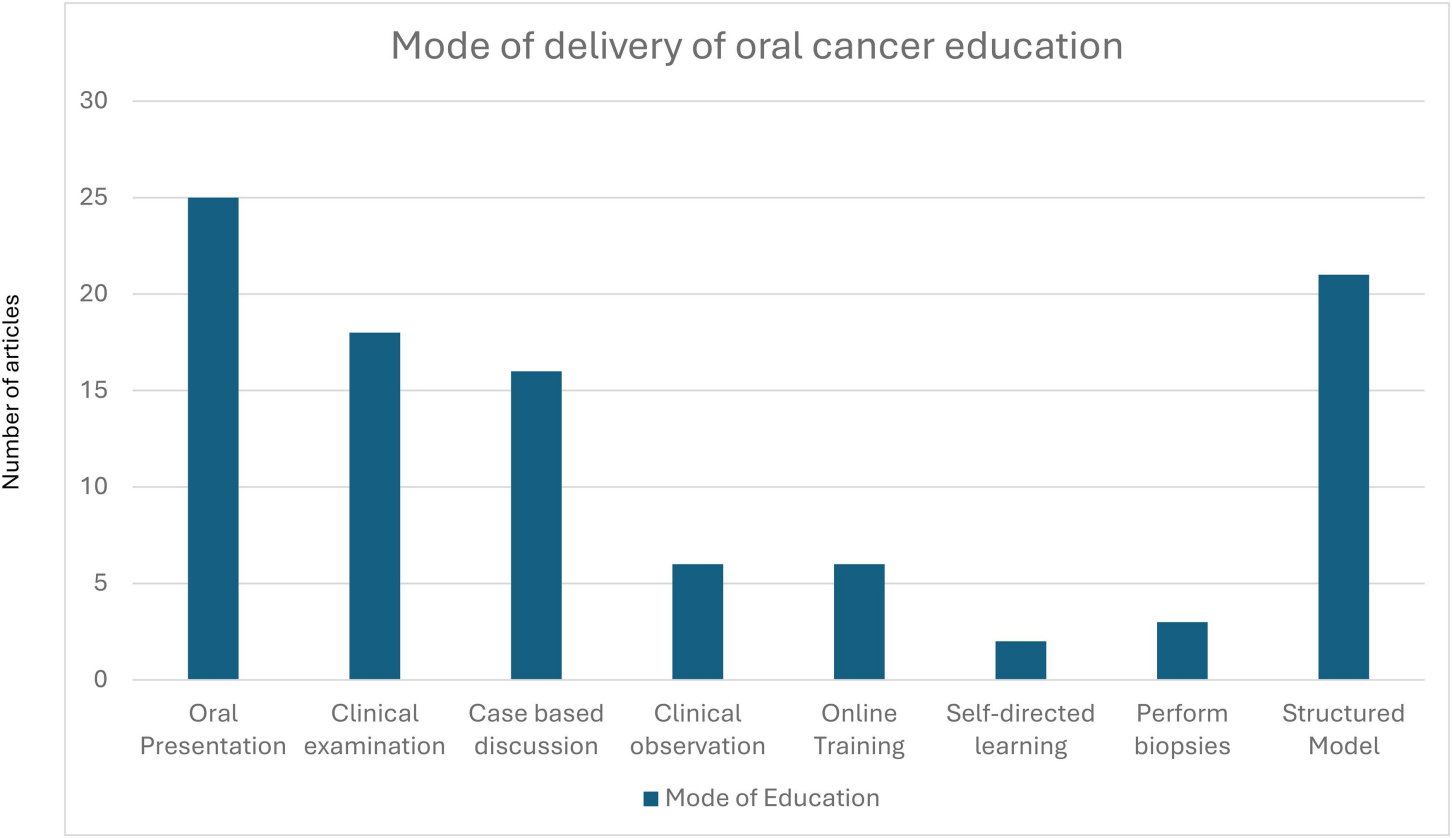
Mode of delivery of oral cancer education.

A variety of teaching methods were utilised across the 39 studies (Figure 3). The most frequently noted methodology involved a form of oral presentation (lecture, or seminar) by a faculty member or specialist on oral cancer or OPMD in a university setting or in continuing education program with 25 studies reporting its use (15, 17, 19, 20, 22–24, 29–31, 34–37, 39–44, 47–50, 53). 18 studies noted the use of clinical examination or hands on instruction as a training method for oral cancer (14, 15, 18, 20, 21, 23–25, 28, 31, 32, 35–37, 40, 43, 50, 52). 16 studies reported the use of case based discussion, case presentations, problem based learning (PBL) models or grand round presentations as a training method for oral cancer (17–19, 22, 27, 29, 31, 35, 36, 38, 42, 43, 47, 50, 51, 53). 6 studies noted the use of clinical exposure, observations or hospital rotations as a training method (15, 18, 20, 28, 33, 43) while 6 studies utilised a form of online or video training (34, 36, 37, 43, 44, 52). Self-directed learning was evaluated in two studies, where participants received oral cancer education through web-based or text- based materials completed at their own pace (29, 48). Only 2 studies, utilised an artificial environment such as oral lesion simulators or a virtual learning object (36, 40). Three studies evaluated the use of performing biopsies as a part of oral cancer training (17, 25, 28). Finally, 21 studies noted the use of a structured educational model such as using a set number of hours for theoretical teaching or hands on training, or a specific teaching model or protocol (17, 19, 22–24, 29, 31, 34–37, 39–45, 47, 48, 50). There were no studies that reported the use of artificial intelligence in oral cancer education.

### Effectiveness of oral cancer education methods

Findings from the studies predominantly noted that any form of oral cancer education intervention, regardless of the mode of delivery utilised was superior in increasing knowledge, confidence and attitudes towards oral cancer in participants compared to baseline (17, 19, 20, 22, 24, 29, 31, 34–52). Oral presentations were the most utilised methodology and were found to be effective in knowledge acquisition when used in combination with other modes of teaching such as hands on training or problem-based learning approach (Table 2). Ilguy et al., was the only study to directly compare lecture-based learning with case-based learning and found that students who were taught with case-based learning methodology had the highest scores in clinical decision making on oral cancer (22). When compared to self-directed teaching only, the use of lectures resulted in significantly higher scores amongst students in oral cancer knowledge (48). Ramanathan et al., utilised a series of five lectures followed by corrective feedback sessions on oral lesions (OralDETECT training programme) and found that the final score on knowledge gained on oral lesions was higher, and significantly associated with the method of administration of the training programme (44). Conversely, Cannick et al., compared the use of PRECEDE-PROCEED framework on tobacco cessation in combination with demonstration of head and neck examination techniques (intervention group) against the pre-determined dental curriculum on oral cancer prevention which included the use of traditional lecture and a PBL approach (control group). The study found that the intervention group demonstrated higher knowledge on oral cancer and tobacco cessation at the 6-months post-test assessment compared to the control group (35). The use of oral presentations through a weekly online course followed by case presentations over 14 weeks noted significant increase in overall mean knowledge on oral cancer screening after each weekly session and at the end of the course (43). Similar results were also noted in Miller et al., and Nethan et al., where oral presentations ranging from 15 to 30 min were delivered online on oral cancer risk factors (41, 42).

Regarding clinical examination, Braun et al., assessed self-efficacy on diagnosis and treatment of oral lesions, and noted that dentists who performed oral mucosal examinations more frequently perceived less difficulty in diagnosing and treating oral lesions (17). Self-efficacy to diagnose and treat oral lesions was also strongly associated with the perception of time spent on theoretical lectures practical learning as adequate (17). Clark et al. compared video only, faculty-led hands-on instruction, and combination of video and hands on instruction on the effectiveness on skill training on theoretical oral cancer education and clinical examination technique. The study found that all students improved in theoretical knowledge and clinical technique. Interestingly, students who received both video and hands on training performed significantly better from students who received video-only training (37). Morelatto et al., also provided theoretical and practical courses through their “Stick your tongue out” campaign to train dentists in detection of oral cancer lesions. The study noted specific improvement in diagnosis of tumours located on the gingiva, floor of mouth and oropharynx post-intervention (50).

Case based discussions or problem based approaches led by experts on oral cancer were found to be effective in improving diagnostic accuracy of oral lesions (19) and knowledge (22) as well as being a preferred teaching method (15, 27). Case based discussions also provided avenues for interprofessional education strategies between undergraduate pharmacy and dentistry students, resulting in increased identification of high-risk patients (51). Interestingly, Seoanne et al., compared case-based discussions methodology against the use of Guidelines published by the Dental Council for referral of oral cancer and found that the proportion of oral cancers that were indicated as urgent referral was significantly higher amongst the participants that utilised the Guidelines (47).

Two studies reported on the efficacy of clinical rotations or observations and found that students who had observed a biopsy or had undergone a clinical rotation with OMFS were more likely to perform a biopsy and refer patients with lesions of the mouth and tongue to the specialty, respectively (20, 28).

Finally, two studies incorporated the use of a virtual learning object or simulator to practice diagnosis of oral lesions. Cardoso et al., accompanied the virtual learning object with PBL methodology and oral presentations by tutors over video while Haresaku et al., provided a 45 min lecture in addition to practical program a simulator. Cardoso et al., noted a positive impact of the teaching methodologies on participants diagnostic ability (36) while Haresaku et al., noted that perceived confidence in oral assessment was higher after the practical program using the simulator when compared to the lecture programme alone (40).

### Challenges in teaching and future direction for oral cancer education

Overall, there is no consensus on content or most appropriate teaching methods in oral cancer education with a need to develop a curriculum that addresses all aspects of oral cancer, as well as improving teaching skills of professors through periodical courses on teaching-learning processes (Table 3). The need for incorporating more thorough hands on training for clinical examination and use of case based discussions was endorsed by several studies (16, 17, 22, 23, 25, 32, 35, 37, 38). Limited studies utilised technology such as online training or virtual reality, with no studies using artificial intelligence. However the studies that did utilise these methods noted the technology to be feasible, and served as a large-scale, best-practice tool for training in oral cancer screening (36, 40, 42, 43). Abbott et al., noted that students graduating from Australian medical schools have little, if any foundational knowledge in oral health, including oral cancer (14). Ahluwalia et al., who evaluated UK GP trainees noted concerns over time constraints and competing priorities within the curriculum (15). Continuing education activities on oral cancer were suggested by some studies to become mandatory as a solution to educational barriers and to maintain satisfactory level of competency (17, 19, 50). The incorporation of multidisciplinary or interprofessional teaching on OPMD and oral cancer with focus of students playing a central role in learning was also recommended by several studies (21, 26, 27, 31, 36, 38, 51).

**Table 3:** Challenges in oral cancer education and future recommendations.

No	Author, Publication Year	Challenges and future direction recommendations
1	Abbott et al. (2018)	Teaching of fundamental oral health to medical students needs to be integrated into all Australian medical school curricula.
2	Ahluwalia et al. (2016)	Oral health training could be improved by access to specialist tutors, e-learning programmes and problem-based-learning sessions. Respondents desired support from other specialists to help improve training but highlighted the need for training sessions to be relevant to GPs. Concerns were raised over time constraints and competing priorities within the curriculum. Some programme directors reflected on the need for trainees learning to be self-directed, emphasising the importance of the training that happened outside the taught programme, in the clinical setting.
3	Ahluwalia et al. (1998)	There is a need to standardise and provide comprehensive training in oral cancer examination techniques and background.
4	Awojobi et al. (2016)	Training in the use of the communication guide was able to successfully address some of the barriers to cancer-related discussions. Dentists still feel that talking about cancer may increase anxiety in patients, which may need attention in future training sessions.
5	Braun et al. (2021)	Continuing education activities on oral cancer encourage dentists to adopt oral mucosal examination regularly, improving self-efficacy to manage oral lesions and detect oral cancer. Engagement in active screening appears to be determinant for improvement of self-efficacy on oral lesion diagnosis and treatment along one's professional career. Future studies should focus on impact of continuing education activities on stage of oral cancer at time of diagnosis, barriers to achieving early detection.
6	Cannick et al. (2007)	PRECEDE-PROCEED theoretical model can be applied as a comprehensive methodology for designing, implementing, and evaluating an oral cancer prevention and early detection intervention. Traditional lecture and PBL approach, reinforced by observing faculty members demonstrate head and neck examination and tobacco cessation can improve detection skills.
7	Cardoso et al. (2022)	A change in the current teaching paradigm is supported: students play a central role, being active and responsible for their own learning, moving away from the teacher centred model in which the teacher is the knowledge older.
8	Carlisle et al. (2021)	Interprofessional education and collaborative committees recommended for universities Include interprofessional requirements within competency standards for both professions, to give IPE more influence
9	Carter et al. (2010)	No consensus on content and most appropriate teaching methods and there is need to develop a curriculum that addresses the important aspects of oral cancer.
10	Clark et al. (2013)	Students performed the clinical examination better with the combination of videos and faculty-led hands-on instructions than either videos alone or faculty-led hands-on instructions alone.
11	Fasbinder et al. (2014)	Focus should be made on clinical issues. Students liked hearing from various experts. Avoid too many separate assignments. Consider introducing interdisciplinary work at beginning of dental course.
12	Gaballah et al. (2022)	Engagement with continuing education activity has improved junior dentists' ability to detect oral mucosal abnormalities, increased their accuracy in stratifying malignant potential of oral lesions. Creating mandatory review course for students before graduation would be a beneficial approach to improve oral cancer knowledge. Regular mandatory educational activities on oral cancer to maintain satisfactory level of competency.
13	Glossop et al. (2024)	Students would benefit from incorporation of structured curriculum for dental concepts.
14	Gomes et al. (2024)	Dental students and dentists lack adequate training for effectively communicating an oral cancer diagnosis. Integration of strategies and skills for delivering challenging diagnoses is essential component within dental curricula. Singular intervention may have potential to significantly enhance readiness to engage critical communication.
15	Hamid et al. (2018)	Exposure to, and awareness of the scope of OMFS and oral cancer teaching is limited in medical schools.
16	Haresaku et al. (2024)	Potential for oral simulators to support training for nurses in oral health education.
17	Heler et al. (2025)	Oral cancer prevention was regarded highest educational priority to implement in curriculum. Interprofessional education in lectures and simulations significantly increased nursing students' overall knowledge.
18	Ilguy et al. (2014)	A case-based approach, which is learner-centred and involves intense interaction among participants, may be more effective in preparing students for deep learning than a lecture-based approach.
19	Lee et al. (2022)	Educational curricula in Korea should provide guidance on comprehensive oral cancer techniques that include both intraoral and extra oral examination and palpation techniques. Provide all dental hygiene students with clinical practice opportunities relevant to oral cancer. Offer continuing education opportunities in oral cancer education to support skill improvement for practicing dental hygienists. Future large-scale studies using objective and specialised tools for improving assessment of oral cancer risk factors and screening findings are needed.
20	LeHew et al. (2009)	There is a need for training on early detection of oral cancer to encourage improving clinical care, but also to reinforce their relevance in routine clinical care.
21	Macedo et al. (2025)	Educational institutes should provide practical training for lymph node examination, performance of biopsies and contact with patients with cancer and lesions with malignant transformation risk.
22	McCann et al. (2005)	Interdisciplinary postgraduate seminars could be used locally by postgraduate students.
23	Miller et al. (2011)	Exposure to a fifteen-minute online module, viewed at the time of their choosing, may be a useful mode of increasing oral cancer education.
24	Morelatto et al. (2022)	Regular free training of dental professionals and the creation of referral networks using teledentistry are crucial for improvement. Recommend continuing with professional training, including a wider spectrum of health professionals, thus favouring preventative attitudes across the primary health care system
25	Nethan et al. (2019)	The time designated during each session for questions and interaction among the participants and the experts facilitated further knowledge enhancement. The ECHO model can be utilized as a feasible, large-scale, best-practice, telementoring tool for training HCPs in oral cancer screening and tobacco cessation.
26	Nethan et al. (2023)	The ECHO model is a promising best-practices tool in reducing the disparity in knowledge and skills regarding oral cancer prevention among dentists across different locations. Removing barriers to receiving knowledge through technology and thereby empowering more HCPs in cancer screening will speed progress towards the overall cancer prevention goal.
27	Ramanathan et al. (2024)	ORALDETECT training programme, via both face-to-face and online delivery methods, is effective in enhancing knowledge and skills with regards to oral cancer and potentially malignant disorders. Slight performance decline via the online delivery method, suggests limitations in sustaining the same level of improvement over time.
28	Ramirez-Amador et al. (2023)	Multidisciplinary approach to teaching OPMD and oral cancer must extend to other dental and nondental courses. Problem based learning and clinical case presentations currently most effective and must be adopted in all OPM curricula. Improve teaching skills of professors through periodical courses on teaching-learning processes.
29	Rankin et al. (1996)	Undergraduate dental students should be required to observe or perform an oral biopsy.
30	Roeschmann et al. (2022)	The integration of a standardized questionnaire seems to be a useful approach to improve lesion description and classification.
31	Roxo-Goncalves et al. (2017)	Distance learning courses may improve the knowledge required for better oral cancer detection, although low motivation and compliance are important obstacles that remain to be overcome.
32	Seoane et al. (2011)	Implementation of a clinical referral guideline for patients with lesions suspicious for malignancy at undergraduate level has proved valuable.
33	Seoane et al. (1999)	Teaching procedures using slides could be useful for training future examiners at recognising oral erythroplakia.
34	Silva et al. (2016)	Percentages of correct answers depends on the which semester students attended teaching. Seventh semester undergraduate students show good knowledge compared to first.
35	Tabrizi et al. (2020)	Interprofessional model allows students to engage with professionals across disciplines and recognise that close collaboration with professionals is fundamental to achieving shared goal of geriatric care. Interprofessional activities should be within the schedule of teaching rather than extracurricular activity. Offering incentives such as certificate of completion, a letter of acknowledgement, or elective course credits should be considered to enhance future participation.
36	Thacker et al. (2015)	Examination techniques taught in dental hygiene programmes need to be more thorough. The capacity to address patient risk factors also needs to be strengthened. Standards need to be kept up to date.
37	Tomar et al	Self-study of assigned reading material may result in lower levels of demonstrated knowledge of oral cancer than more traditional lecture-based teaching.
38	Uti et al. (2006)	Increased emphasis on the early signs and symptoms of oral malignancy and increased involvement in examination and biopsies of malignant and premalignant lesions by students.
39	Walsh et al. (2013)	Lecture format significantly increased performance of both oral cancer screening and tobacco cessation counselling. Use of lecture format session for large groups may be an efficient and cost-effective public health method of teaching dental professionals about the latest science

## Discussion

This scoping review aimed to examine the current methodologies utilised in teaching oral cancer and OPMDs to health care professionals and students. Studies included in this review demonstrate that there is currently a wide variety of methodologies used, varying from oral presentations (lectures, seminars), hands on training of head and neck clinical examination, case-based discussions and PBL approaches, hospital rotations, performing oral biopsies and varying structured educational models, with currently no consensus on the most effective teaching method.

Interestingly, the most utilised mode of education was oral presentations, although it appeared to have variability in its effectiveness. Traditionally, the use of lectures has been one of the most utilised teaching methods in universities; however, its usefulness is often questioned as it is dependent on how engaging the speaker is, the size of the group and students' listening, attendance, and note-taking skills (54). There is also now a greater understanding of how active listening can benefit from other self-directed cognitive and emotional processes, with interpersonal communication skills further mobilising participants (55). As such, there has been a greater push to renew the current format of lecture-based teaching with suggestions to increase interactive learning and utilise multimedia material (54, 55). These findings are also reflected in the results noted by Haresaku et al., and Ramanathan et al., where lecture based teaching was accompanied by a practical program using oral simulators (multimedia use), or followed by corrective feedback sessions (interactive learning) respectively, noting significant improvement in knowledge gained on oral cancer (40, 44). While oral presentations have their place in oral cancer education, university curriculums and continuing education programs should consider its use alongside a complimentary training method which encourages greater participant interaction.

The importance of hands-on training in head and neck clinical examination was reinforced by multiple studies, including the need to improve the quality of training (16, 23, 25, 32, 33, 37, 38). Most recently, Macedo et al. noted that 84.4% of dental students believed their lymph node palpation training was inadequate, while Thacker et al., found that only 37% of dental hygiene programs taught palpation of tonsils and tonsillar pillars (25, 32). Alarmingly, Abbott et al., reported that no medical schools in Australia provided hands-on oral health training, with Ahluwalia et al., also noting that 71.2% of UK GP training programmes do not provide any structured oral health education for trainees (14, 15). Those that provided oral cancer education reported that only 28.6% of medical schools examined all anatomical areas of the oral cavity, highlighting an urgent need for improving head and neck clinical examination within medical programs and amongst medical professionals (16). With regards to hands-on training as a methodology, observing an expert demonstrating proper head and neck examination techniques compared to traditional lecture and PBL approaches appeared to be more effective in oral cancer prevention and early detection (35). Complementing faculty-led hands-on instruction with video instruction appeared to show greater improvement than hands-on training alone, suggesting that a mixed methods approach may be most effective in teaching clinical examination skills (37). Further, Ramirez-Amador et al., emphasised the importance of teaching and practicing oral examinations from 1st year of undergraduate levels, and throughout the study courses to ensure competency (27). Given that early detection of oral cancer and OPMD is contingent on a comprehensive oral examination, it is imperative that current medical and dental programs as well as continuing education programs review their present curriculum and ensure hands on training in head and neck examination is appropriately established.

Critical thinking is essential to health practice, enabling healthcare professionals to analyse complex information, make informed decisions and provide high-quality care. This holds particularly true within the context of oral cancer and OPMDs, where clinical decision making has direct impact on patient outcomes. Numerous studies have explored the impact of PBL on critical thinking skills in medical education, with a recent systematic review and meta-analysis demonstrating PBL to be significantly more effective than conventional teaching methods in enhancing critical thinking skills among medical students (56). Findings from the scoping review also support this, with PBL methodologies utilised by Cardoso et al., Gaballah et al., and Ilguy et al., showing a 13.4% increase in correct classification of oral lesions compared to baseline, improvement in overall diagnostic accuracy, and clinical decision making skills, respectively (19, 36). Greater use of PBL and case-based learning was also collectively endorsed by professors involved in teaching oral medicine during a workshop analysing and discussing the main challenges of teaching OPMDs and oral cancer (27). Case-based learning and PBL approaches also allow greater spread of knowledge across professions, proving to be a sound approach to improving multidisciplinary care. Interestingly, only a few studies have described oral cancer education through interprofessional training (29, 31, 42, 51), even though many studies supported this idea (21, 27, 31, 51). There is now considerable evidence showing that allied health professionals including community pharmacists, nurses, and community health care workers are frequently consulted regarding oral health concerns (57, 58). It is imperative that oral cancer education delivery utilises case-based interprofessional training methods as it is fundamental to achieving the shared goal of reducing the burden of oral cancer.

Over recent years technological innovations and advancements have significantly altered the landscape of dental care and education, embracing technologies such as intra oral scanners, 3D imaging, electronic health records and computer-aided manufacturing (CAD/CAM) systems (59). Further, the emergence of IVR technology as a tool to simulate clinical situations has also been utilised and has shown to provide hand skill transfer to the real world and increasing user engagement and cognition (60, 61). Surprisingly, oral cancer education appears to be behind in adopting such technologies, with only two studies reporting the use of virtual simulations and none reporting the use of artificial intelligence (36, 40). It appears that utilisation of technologies in oral cancer education has been primarily limited to online lectures and modules (29, 42–44, 52). Although there is evidence that knowledge on oral cancer can be increased via online series, Ramanathan et al., found that this delivery style may not be as useful for long-term knowledge retention compared to face-to-face education (44). Further studies are required to adopt evolving digital technologies and to evaluate their effectiveness within the context of oral cancer education.

Finally, the greatest challenges within the delivery of oral cancer education appear to be time constraints. This is reflected in the alarmingly low number of hours being spent on oral cancer education, particularly amongst medical schools (14, 17–19, 24, 34, 36, 39–43, 46, 48, 49). It has been shown that there is a direct correlation between a clinician's ability and confidence in detecting and diagnosing oral lesions frequently, and the perception of time spent on theoretical knowledge, practical training and attendance of continuing education programs (17). Increasing the amount of time spent on oral cancer education within universities and the provision of more (perhaps free) continuing education programs on oral cancer may be a simple and effective solution to improving early detection and diagnosis of oral cancer and OPMD.

This scoping review has some limitations that should be acknowledged. First, the heterogeneity of outcome measures across the included studies limits the ability to compare findings directly. The restriction to English-language publications may have also resulted in exclusion of relevant studies published in other languages. Finally, the predominance of cross-sectional study designs among the included literature restricts the ability to infer associated relationships.

## Conclusion and recommendations

The results of this scoping review highlight the wide range of methodologies currently used to deliver oral cancer education. Although consensus on the most effective mode of delivery is lacking, findings across all included studies consistently demonstrate that any form of oral cancer education intervention is superior to none. Notably, there is a clear need to expand the provision of hands-on clinical training, particularly in head and neck examination skills, across all health care university programs as well as in continuing professional development activities.

In addition, case-based and learner-centred approaches, which promote interaction and active engagement among participants, appear more effective in fostering deeper learning and enhancing critical thinking skills than traditional didactic lectures alone. Emerging educational technologies, such as virtual reality has seen limited use in oral cancer education, while artificial intelligence remains largely unutilised and provides avenues for further investigation.

Finally, this review underscores an urgent need for universities, particularly dental and medical schools, to increase the time allocated to oral cancer education. Strengthening curricular emphasis in this area is essential to improving professional knowledge and, ultimately, enhancing the early detection and diagnosis of oral cancer.

## Supplementary material

Supplementary Table 1 Grey literature search strategy.

Supplementary Table 2 Exclusion list of Full-text articles.
