# Supplementary material for: How oral cancer is taught: a scoping review
Source: Front Oral Health. 2026 Jul 30;7:1841566. doi: 10.3389/froh.2026.1841566 (PMC13470247; doi:10.3389/froh.2026.1841566)
Supplement: Supplementary file 1 [file Table1.docx]

Supplementary Table 1: Grey literature search strategy

| **Search engine** | **Search strategy** | **# of items screened** |
| --- | --- | --- |
| Google Scholar | - Search: Mode of delivery of oral cancer education - Inclusion and exclusion criteria applied to screening | - 50 items screened - 48 not relevant to OPMD/OSCC education in health professionals - 2 Duplicates |
| Google Scholar | - Search: Delivery of oral cancer education - Inclusion and exclusion criteria applied to screening | - 50 items screened - 47 not relevant to OPMD/OSCC education in health professionals - 3 Duplicates |
