# Supplementary material for: How oral cancer is taught: a scoping review
Source: Front Oral Health. 2026 Jul 30;7:1841566. doi: 10.3389/froh.2026.1841566 (PMC13470247; doi:10.3389/froh.2026.1841566)
Supplement: Supplementary file 2 [file Table2.docx]

Supplementary Table 2: Exclusion list of Full-text articles

| **Reason for exclusion** | **Article Citation** |
| --- | --- |
| **Did not include eligible participants** | 1. Noro LRA, Oliveira, Gurgel BCV, Araujo PH. Validation of the criteria matrix about the generalist's education in Dentistry Undergraduate Courses. Pesqui Odontol Bras. 2021;35. doi:10.1590/1807-3107bor-2021.vol35.0089 |
| **Did not include mode of delivery for oral cancer/OPMD education** | 1. Poelman MR, Brand HS, Asadi M, Remmelzwaal S, Jager DH, de Visscher JG. Knowledge of diagnosis and management of selected oral mucosal lesions among dentists in The Netherlands. Med Oral Patol Oral Cir Bucal. 2023;28(4):e362-e370. doi:10.4317/medoral.25774 2. Ali K, Cockerill J, Zahra D, Qazi HS, Raja U, Ataullah K. Self-perceived preparedness of final year dental students in a developing country: a multi-institution study. Eur J Dent Educ. 2018;22(4):e745-e750. doi:10.1111/eje.12389 3. Berkowitz O, Brisotti MF, Gascon L, Henshaw M, Kaufman LB. The impact of an interprofessional oral health curriculum on trainees. J Physician Assist Educ. 2017;28(1):2-9. doi:10.1097/JPA.0000000000000104 4. Jafer M, Crutzen R, Jafer A, van den Borne B. What do dental college clinicians know about oral cancer and its risk factors? An assessment among final year students, interns and faculty members in Saudi Arabia. J Clin Exp Dent. 2018;10(9):e908-e913. doi:10.4317/jced.55168 5. Maybury C, Horowitz AM, Goodman HS. Outcomes of oral cancer early detection and prevention statewide model in Maryland. J Public Health Dent. 2012;72 Suppl 1:S34-S38. doi:10.1111/j.1752-7325.2012.00320.x 6. Decuseara G, MacCarthy D, Menezes G. Oral cancer: knowledge, practices and opinions of dentists in Ireland. J Ir Dent Assoc. 2011;57(4):209-214. 7. Macedo DR, Maciel EF, Rodrigues JF, Cardoso SV, de Oliveira GJPL, Soares PBF. Assessment of dental students' knowledge and attitudes about the epidemiologic and clinical characteristics of oral cancer: implications for other middle-income countries. J Cancer Educ. 2025;40(3):432-440. doi:10.1007/s13187-024-02529-3 8. Schiavo-Di Flaviano V, Gonzalez-Navarro B, Martin Carreras-Presa C, Jane-Salas E, Lopez-Lopez J. Attitude of general dental practitioners, oral surgeons, and dentistry students towards oral lesions and oral biopsy. Med Oral Patol Oral Cir Bucal. 2025;30(1):e31-e40. doi:10.4317/medoral.26804 9. Antoranz A, Navarrete N, Casanas E, Munoz-Corcuera M. Oral cancer awareness among dental students in a private university setting. Eur J Dent Educ. 2024;28(3):718-723. doi:10.1111/eje.13000 10. Brucoli M, Boffano P, Pezzana A, et al. The potentialities of the Anatomage Table for head and neck pathology: medical education and informed consent. Oral Maxillofac Surg. 2020;24:229-234. 11. Burzynski NJ, Rankin KV, Silverman S, Scheetz JP, Jones DL. Graduating dental students’ perceptions of oral cancer education: results of an exit survey of seven dental schools. J Cancer Educ. 2002;17(2):83-84. doi:10.1080/08858190209528804 12. Shrestha A, Marla V, Shrestha S, Agrawal D. Awareness of undergraduate dental and medical students towards oral cancer. J Cancer Educ. 2017;32(4):778-783. doi:10.1007/s13187-016-1023-4 13. Jayasinghe RD, Sherminie LP, Amarasinghe H, Sitheeque MA. Level of awareness of oral cancer and oral potentially malignant disorders among medical and dental undergraduates. Ceylon Med J. 2016;61(2):77-79. doi:10.4038/cmj.v61i2.8289 14. Bakr MM, Skerman E, Khan U, George R. Oral cancer: an evaluation of knowledge and awareness in undergraduate dental students and the general public. Oral Health Prev Dent. 2016;14(5):403-411. doi:10.3290/j.ohpd.a36471 15. Hassona Y, Scully C, Shahin A, Maayta W, Sawair F. Factors influencing early detection of oral cancer by primary health-care professionals. J Cancer Educ. 2016;31(2):285-291. doi:10.1007/s13187-015-0823-2 16. Langelier MH, Glicken AD, Surdu S. Adoption of oral health curriculum by physician assistant education programs in 2014. J Physician Assist Educ. 2015;26(2):60-69. doi:10.1097/JPA.0000000000000024 17. Kujan O, Alzoghaibi I, Azzeghaiby S, Altamimi MA, Tarakji B, Hanouneh S, et al. Knowledge and attitudes of Saudi dental undergraduates on oral cancer. J Cancer Educ. 2014;29(4):735-738. doi:10.1007/s13187-014-0647-5 18. Pentenero M, Chiecchio A, Gandolfo S. Impact of academic and continuing education on oral cancer knowledge, attitude and practice among dentists in North-Western Italy. J Cancer Educ. 2014;29:151-157. doi:10.1007/s13187-013-0562-1 19. Silverman S Jr, Kerr AR, Epstein JB. Oral and pharyngeal cancer control and early detection. J Cancer Educ. 2010;25(3):279-281. doi:10.1007/s13187-010-0045-6 20. Brocklehurst PR, Baker SR, Speight PM. A qualitative study examining the experience of primary care dentists in the detection and management of potentially malignant lesions. 2. Mechanics of the referral and patient communication. Br Dent J. 2010;208(2):E4. doi:10.1038/sj.bdj.2010.55 21. Munoz N, Touger-Decker R, Byham-Gray L, Maillet JO. Effect of an oral health assessment education program on nurses' knowledge and patient care practices in skilled nursing facilities. Spec Care Dentist. 2009;29(4):179-185. doi:10.1111/j.1754-4505.2009.00084.x 22. Nicotera G, Di Stasio SM, Angelillo IF. Knowledge and behaviors of primary care physicians on oral cancer in Italy. Oral Oncol. 2004;40(5):490-495. doi:10.1016/j.oraloncology.2003.10.001 23. Clovis JB, Horowitz AM, Poel DH. Oral and pharyngeal cancer: practices and opinions of dentists in British Columbia and Nova Scotia. J Can Dent Assoc. 2002;68(7):421-425. 24. Jullien JA, Downer MC, Speight PM, Zakrzewska JM. Evaluation of health care workers' accuracy in recognising oral cancer and pre-cancer. Int Dent J. 1996;46(4):334-339. |
| **Non-English** | 1. Onderwijs. Training in oral cancer and precancer; guidelines from the Commission of the European Community. (in Dutch) Ned Tijdschr Tandheelkd. 1989;96(4):159-161. |
